# Modeling the Converse Magnetoelectric Effect in the Low-Frequency Range

**DOI:** 10.3390/s24010151

**Published:** 2023-12-27

**Authors:** Mirza Bichurin, Oleg Sokolov, Sergey Ivanov, Viktor Leontiev, Vyacheslav Lobekin, Gennady Semenov, Yaojin Wang

**Affiliations:** 1 Yaroslav-the-Wise Novgorod State University, 173003 Velikiy Novgorod, Russia; o-v-sokolov@mail.ru (O.S.); sivanovvad@mail.ru (S.I.); viktor.leontev@novsu.ru (V.L.); slavalobekin@gmail.com (V.L.); gennady.semenov@novsu.ru (G.S.); 2 School of Materials Science and Engineering, Nanjing University of Science and Technology, Nanjing 210094, China; yjwang@njust.edu.cn

**Keywords:** magnetoelectric effect, direct magnetoelectric effect, converse magnetoelectric effect, magnetoelectric composite, magnetoelectric coefficient, electromechanical resonance, resonance mode, bimorph structure

## Abstract

This article is devoted to the theory of the converse magnetoelectric (CME) effect for the longitudinal, bending, longitudinal-shear, and torsional resonance modes and its quasi-static regime. In contrast to the direct ME effect (DME), these issues have not been studied in sufficient detail in the literature. However, in a number of cases, in particular in the study of low-frequency ME antennas, the results obtained are of interest. Detailed calculations with examples were carried out for the longitudinal mode on the symmetric and asymmetric structures based on Metglas/PZT (LN); the bending mode was considered for the asymmetric free structure and structure with rigidly fixed left-end Metglas/PZT (LN); the longitudinal-shear and torsional modes were investigated for the symmetric and asymmetric free structures based on Metglas/GaAs. For the identification of the torsion mode, it was suggested to perform an experiment on the ME structure based on Metglas/bimorphic LN. All calculation results are presented in the form of graphs for the CME coefficients.

## 1. Introduction

The continuous development of technology requires constant involvement in the process of materials with new multifunctional properties. One of these materials is a composite material with a magnetoelectric (ME) effect [1,2,3]. A large number of studies are already known on these materials in connection with the prospects for their use in the development of new electronic devices [4]. There is the direct ME effect (DME) and converse ME effect (CME) [5], where the direct effect is characterized by the induction of an output electrical voltage in the ME composite under the influence of a magnetic field, and the converse effect is the excitation of magnetization under the influence of an external electric field. It should be noted that the converse effect is often referred to in the literature as inverse. The DME effect has already been studied in sufficient detail [5,6], and on its basis, a number of promising electronic devices [7,8,9,10] have been proposed. Relatively few works have been devoted to the CME effect. The first studies of the CME effect were associated with the microwave ME effect, which consists of a shift in the ferromagnetic resonance (FMR) line in the ME structure under the influence of an external electric field [11,12,13,14,15] and the further use of this effect to create tunable microwave devices [5,6]. In recent years, research interest in the CME effect in the low-frequency region has increased. This is due to the great interest in the design of magnetic field sensors [16,17,18] and low-frequency ME antennas [19,20,21,22] and the possibility of their use in spintronics [23,24,25,26,27,28] and ME memory devices [29,30,31]. The purpose of this article is a brief analysis of the works on the CME effect and to obtain expressions for CME coefficients for the low-frequency region, including the electromechanical resonance (EMR) modes: longitudinal, bending, longitudinal-shear, and torsional.

Below is a brief overview of the work on the low-frequency CME effect in composites. In one of the first works, Jia et al. [32] theoretically and experimentally studied the CME effect in the Terfenol-D/PMN-PT structure and obtained an ME coefficient of 105 mG/V at a bias field of 170 Oe. A linear relationship was found between the measured magnetic induction and the applied alternating electrical voltage in the range of 50–160 V. In [33,34,35,36,37,38,39,40,41,42,43,44], the authors examined the features of the CME effect in various structures. In [33], the measurements of CME effects were conducted in the trilayers of PZT/Ni/PZT. A pickup coil wound around the sample was used to measure the CME effect due to the change in the magnetic induction in Ni. The measured static magnetic-field dependence of the ME voltage has been attributed to the variation in the piezomagnetic coefficient for Ni. The frequency dependence of the CME effect showed a resonance character due to radial acoustic modes in PZT. Chen et al. [34] investigated the CME effect in a transition metals-based ferromagnetic-shape memory alloy/piezoelectric ceramic-laminated composite. The strong CME effect was observed at room temperature over a broad bandwidth, under weak magnetic bias and an electric field. The authors of [35] studied the CME effect in a two-layer composite consisting of piezoelectric PZT and ferrite MZF plates. Dependences of the magnetic induction variation Bm on the amplitude and frequency of the AC electric field, as well as on magnetic field strength, temperature, and composition for layered PZT/MZF composite, have been determined. It is shown in [36] that the voltage transformation coefficient depends not only on the CME coefficient and the number of turns of the inductor but also on the relative orientation of the magnetic field and the geometric dimensions of the sample. The CME effect was studied [37] in a layered composite Metglas/PMN-PT by the induction method at various frequencies. A large CME coefficient of 3.05 G/V was observed at a resonant frequency of 76.5 kHz in a weak bias magnetic field of 50 Oe. The CME coefficient of the heterostructure is practically constant and has a relatively high value in a wide frequency range of 1–64 kHz. Xuan et al. [38] investigated the CME effect in Metglas/PZT, and the highest value of the CME coefficient was 4.18 G/V at the resonant frequency of 103 kHz in a low bias field of 45 Oe. In addition, the authors presented data on the known CME coefficients, where the highest value was 18.6 G/V at a frequency of 47 kHz, obtained for a three-layer Ni-Mn-Ga/PMN-PT/Ni-Mn-Ga structure. In [39], using the design of a Rosen-type piezoelectric transducer and a Terfenol-D plate with a Terfenol-D thickness of 0.7 mm, a gigantic CME coefficient of 35.7 G/V was obtained. Lu et al. [40] demonstrated the equivalence of the DME effects and the CME ones in two-phase systems consisting of piezoelectric and magnetostrictive materials. This was achieved by reformulating Maxwell’s relation in terms of the effective electric and magnetic dipole moments of the system and comparing the coupling forces at the same electric and magnetic DC biases. In [41], a dispersed composite consisting of 65 mol. % Na_0.5_Bi_0.5_TiO_3_ and 35 mol. % CoFe_2_O_4_ and its structure, microstructure, ferroelectric, magnetostrictive, magnetic, and direct/converse magnetoelectric properties were studied. The composite showed different magnetization behaviors under electrically polarized and unpolarized conditions. The percentage change in magnetization due to polarization is approximately 15% at a magnetic field of 500 Oe. Bilayer modeling of DME and CME coefficients for longitudinal modes in the EMR region has been considered and presented in [45]. As an example, specific cases of bilayers of cobalt ferrite/PZT and nickel/PZT bilayers were studied. In [45], the DME and CME in Metglas/LN and Metglas/PMN-PT trilayers have been studied in the range from 20 Hz to 0.4 MHz. It is shown that, as a result of the difference in piezoelectric constants, the CME in the structure with PMN-PT is significantly greater (~2 orders of magnitude) than with LN. The article by [46] is devoted to the study of the linearity of the CME effect and improving sensitivity in the low-frequency range. Kalgin et al. [43] researched the CME effect in the ME structures consisting of a layer of Terfenol-D ferromagnetic powder, epoxy adhesive, and a polarized piezoelectric layer of PZT. The dependences of the CME effect on the frequency and strength of the electric field, the strength of the constant magnetic field, the thickness of the ferromagnetic layer, the average size of Terfenol-D granules in the ferromagnetic layer, and temperature have been established. The conditions for obtaining the maximum CME effect are analyzed. In [44], the authors proposed a new bilayer-laminated ME composite consisting of magnetostrictive Ni and Terfenol-D plates and a piezoelectric PZT plate. The bias magnetic field and the electric field frequency dependences of the CME coefficient were investigated. It was shown that the Ni/Terfenol-D/Ni/PZT exhibits a large CME coefficient of 6.2 × 10^−7^ s/m at the electric field frequency of 42 kHz under a low bias magnetic field of 230 Oe. Along with the listed works, which present a measurement technique and provide estimates of the CME effect, there are known, as noted earlier, works that describe the first practical applications of the CME effect. Chu et al. [16] studied the potential of an electrically driven bulk magnetic field sensor based on the CME effect. It was experimentally found that a limit of detection of 115 pT for a magnetic field of 10 Hz and 300 pT for a magnetic field of 1 Hz was achieved by exciting the ME laminate by 1 V without any bias field. In this case, the power consumption for the ME laminate is only 0.56 mW, which is much lower compared to tens of milliwatts (10–100 mW) for optically pumped or fluxgate sensors and also shows advantages over conventional ME magnetic field sensors based on the DME effect with a current pump. In [17], the authors presented the equivalent magnetic noise of a multi-push–pull magneto (Elasto) electric sensor, implemented as a magnetometer with phase modulation circuitry. Based on the constitutive equations and the Nyquist theorem, they have determined the expected sensitivities and the equivalent magnetic noise for the phase modulation and provided a comparison with experimental results. For measurement applications, the authors [18] investigated the CME effect on a thin-film silicon cantilever of a size of 25 mm × 2.45 mm × 0.35 mm using a piezoelectric AlN film and a magnetostrictive FeCoSiB film, each 2 μm thick. The measured sensitivity at the resonant frequency of 515 kHz increases nearly linearly with a carrier signal amplitude, reaching 64 kV/T at 160 mV. The DC detection limit is 210 pT/Hz1/2, and also about 70 pT/Hz1/2 at 10 Hz without magnetic field bias. The obtained high characteristics are of great interest for biomagnetic applications. Recently, research into ME transmitting antennas based on the CME effect in the low-frequency range has been very active [19,20,21] since their creation will significantly reduce the size and ensure effective underwater and underground communications. Yao et al. [19] proposed a bulk acoustic wave-mediated multiferroic antenna structure. Its potential for efficient radiation of electromagnetic waves is evaluated by analytically deriving the lower bound of its Q factor. A one-dimensional multiscale finite-difference time-domain technique was developed to predict the bilateral, dynamic coupling between acoustic waves and electromagnetic waves. Dong et al. [20] presented a novel, very low-frequency communication system using one pair of ME antennas. With 80 V driving voltage, the power consumption of the ME transmitter has been measured as 400 mW at the maximum communication distance of 120 m. In [21], Y. Wang et al. investigated a ME antenna based on mechanical resonance made by MEMS technology. The ME resonant disk structure with a diameter of 70 μm and a thickness of 1 μm with SiO_2_/Cr/Au/AlN/Cr/Au/FeGaB-stacked layers was prepared on a 300 μm silicon wafer and showed a giant ME coefficient of 2.928 kV/cm/Oe in resonance at 224.1 kHz. The article by [22] is devoted to assessing the CME effect within the framework of a two-dimensional multifield communication model for an acoustically controlled antenna. To compare the results obtained in our paper, it is necessary to clarify the details of the calculation in [22]. In addition to the use of the CME effect in sensors and antennas, this effect, as noted, is beginning to be widely explored for applications in spintronics [23,24,25,26,27,28] and ME memory devices [29,30,31]. Zavaliche et al. [23] used piezoelectric force microscopy and magnetic force microscopy to locally image the coupled piezoelectric-magnetic switching in epitaxial ferroelectric BiFeO_3_-ferrimagnetic CoFe_2_O_4_ columnar nanostructures. They presented a perpendicular CME susceptibility of 1.0 × 10^−2^ G cm/V induced by an electric field. In [24], the authors showed that the magnetization of the NiFe/CoFe film on PZT substrate changes by around 6% at the low voltage of 60 V, applied to PZT substrate and is up to two times larger at 800 V in an external magnetic field of 50 Oe. The use of voltage also allows the reversible adjustment of the magnetization orientation in ferromagnetic layers. Chopdekar et al. [25] investigated a (011)-oriented ferroelectric PMN-PT substrate with an epitaxial ferromagnetic (La,Sr)MnO_3_ film and showed that the electric field pulses of 6 kV/cm induce large, reversible, and bistable remanent strains. The authors demonstrated that an electric field pulse can be used to ‘set’ and ‘reset’ the magnetic anisotropy orientation and resistive state in the film for the electric-field manipulation of nanostructures at room temperature. Spaldin and Ramesh in [26] discussed that the change in magnetic properties by an electric field in ME multiferroic materials has driven significant research activity, with the goal of realizing their transformative technological potential. Ghidini et al. [27,28] investigated the Ni films coupled via strain to ferroelectric substrates of BTO and PMN-PT. The authors studied the shear strain-induced CME effects in Ni thin films on PMN-PT and the reversible switching of magnetization in Ni films on barium titanate. Eerenstein et al. [29] showed electrically induced giant, sharp, and persistent magnetic changes (up to 2.3 × 10^−7^ sm^−1^) in ferromagnetic 40 nm LSMO films on 0.5 mm ferroelectric BaTiO_3_ substrate. Observed X-ray diffraction confirms strain coupling via ferroelastic BaTiO_3_ domains. Hu and Nan [30] presented a phenomenological model for the electric-field-induced reorientation of the magnetic easy axis in ferromagnetic Fe, CFO, Ni, Fe_3_O_4_/ferroelectric PZN-PT, BTO, and PZT-layered heterostructures. The authors showed that as the applied electric field increases, the easy axes tend to switch the directions in CFO and Ni films and highlight that this effect has great potential in prototype electrically controlled magnetic recording devices. Wang et al., in their review [31], examined the theoretical and experimental possibilities of an electric field for controlling magnetism through strain coupling in CoFeB/PMN–PT and discussed the problems and prospects of using this method to create ME memory. The authors showed that investigating the effect of low-frequency CME is useful for future research.

The structure of the article is as follows. The purpose of the article is indicated in the introduction. Section 2 and Section 3 are devoted to the consideration of the longitudinal and bending modes in symmetric and asymmetric ME structures. Section 5 and Section 6 describe the longitudinal—shear and torsional modes—in such structures. Note that in Section 4 and Section 8, as a special case, the quasi-static regime is also considered. A discussion of the calculation method specifics used is included in Section 9. Section 10 presents the conclusions of this paper.

## 2. EMR Longitudinal Mode

### 2.1. Symmetric Structure

#### 2.1.1. Free Plate

We accept the alternating magnetic field intensity in the ME composite’s magnetostrictive phase to be zero according to [32]: $h_{1}=0$.

The full thickness of the ME composite is

(1) t=tp+2tm,

where tp and tm are the piezoelectric and magnetostrictive phase thicknesses.

The magnetostrictive and piezoelectric volume fractions are

**Figure 1:**
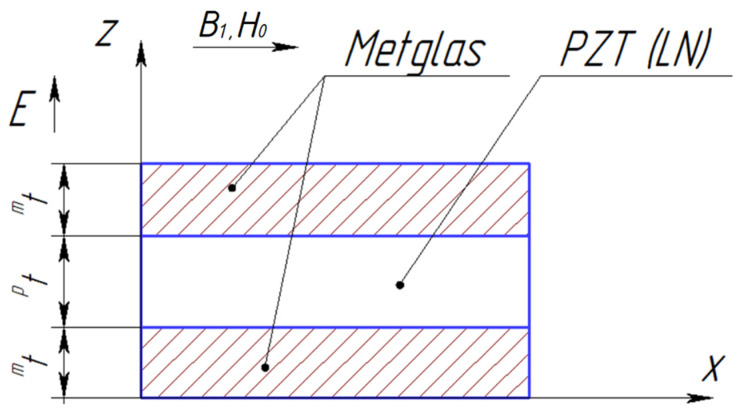
Symmetric ME composite scheme.

(2) νm=2tmtνp=tpt,

Figure 1 shows the ME composite’s structure for calculating the longitudinal mode of the CME effect:

The strain tensor longitudinal component for magnetostrictive and piezoelectric phases is

(3) S1=Sp1=Sm1=∂Ux∂x,

where $U_{x}$ is the longitudinal displacement.

The material equation of the piezoelectric phase is

(4) Tp1=S1sp11−d31E3sp11,

where $E_{3}$ is the alternating electric field intensity applied to the piezoelectric phase of the ME composite, sp11 is the piezoelectric phase’s elastic compliance coefficient, and $d_{31}$ is piezoelectric coefficient.

The material equations of the magnetostrictive phase are

(5) mT1=YmS1−q11h1,

(6) Bm1=μμ0h1+q11mT1,

where Ym is the Young’s modulus of the magnetostrictive phase, $q_{11}$ is the piezomagnetic modulus, and μ is the magnetostrictive phase’s permeability.

Equations (5) and (6) take the form for $h_{1}=0$:

(7) mT1=YmS1,

(8) Bm1=q11mT1,

Substitute Equation (7) into Equation (8):

(9) Bm1=q11YmS1,

The longitudinal component of the ME composite stress tensor is:

(10) T1=νmTm1+νpTp1=c11S1−νpd31sp11E3,

where

(11) c11=νpsp11+νmYm,

The motion equation for deformations of ME composite is [47]

(12) $$\rho \frac{\partial^{2}U_{x}}{\partial t^{2}}=\frac{\partial T_{1}}{\partial x},$$

Substitute Equation (10) into Equation (12):

(13) $$-\rho \omega^{2}U_{x}=c_{11}\frac{\partial^{2}U_{x}}{\partial x^{2}},$$

where ω is the angular frequency of the ME composite’s deformations, and the composite effective density is

(14) ρ=νmρm+νpρp,

where ρp and ρm are the piezoelectric and magnetostrictive phase densities.

The solution to the motion equation for deformations of ME composite is

(15) $$U_{x}=A\cos\left(kx\right)+B\sin\left(kx\right),$$

where the wave number is

(16) $$k=\sqrt{\frac{\rho }{c_{11}}}\omega ,$$

Then, the longitudinal component of the strain tensor has the form:

(17) $$S_{1}=\frac{\partial U_{x}}{\partial x}=\left(B\cos\left(kx\right)-A\sin\left(kx\right)\right)k.$$

The equilibrium conditions for a free ME composite are

(18) $$\begin{array}{l} {\left.T_{1}\right|}_{x=-\frac{L}{2}}=0\\ {\left.T_{1}\right|}_{x=\frac{L}{2}}=0 \end{array},$$

We obtained a linear inhomogeneous equation system for two unknowns, A and B, by substituting Equations (17) and (10) into Equation (18), and found two unknowns A and B from this equation system and substituted them into Equation (17). Then, the obtained expression for the longitudinal component of the strain tensor is substituted into Equation (9), and we find the alternating magnetic induction occurring in the magnetostrictive phase during the CME effect:

(19) Bm1=q11Ymνpd31E3cos(kx)sp11c11cosη,

where $\eta =\frac{kL}{2}$.

The alternating magnetic induction in the ME composite averaged over the magnetostrictive phase volume during the CME effect is as follows:

(20) B¯m1=1tL∫−L2L2Bm1dx∫0tm2dz=2tmq11Ymνpd31E3tgηsp11c11ηt,

The CME coefficient is

(21) αinv=B¯m1E3=2q11tmYmνpd31tgηsp11c11ηt,

The fundamental resonant frequency for this case is

(22) $$f_{r}=\frac{1}{2L}\sqrt{\frac{c_{11}}{\rho }},$$

#### 2.1.2. Rigid End of the Plate

Boundary conditions for the case when the left end of the ME composite is rigidly fixed, and the right one is free:

(23) $$\begin{array}{l} U_{x}\left(0\right)=0\\ T_{1}\left(L\right)=0 \end{array},$$

Substitute Equations (10) and (15) into Equation (23):

(24) A=0c11BcoskL−AsinkLk−νpd31sp11E3=0,

Then, we obtain

(25) A=0B=νpd31E3sp11c11kcoskL,

Completely analogous to the case with a free ME composite, we find

(26) Bm1=q11Ymνpd31E3cos(kx)sp11c11coskL.

The alternating magnetic induction in the ME composite averaged over the magnetostrictive phase volume during the CME effect is

(27) B¯m1=1tL∫0LBm1dx∫0tm2dz=2tmq11Ymνpd31E3tgkLsp11c11kLt,

The CME coefficient is

(28) αinv=B¯m1E3=2tmq11Ymνpd31tgkLsp11c11kLt,

The fundamental resonant frequency for this case is

(29) $$f_{r}=\frac{1}{4L}\sqrt{\frac{c_{11}}{\rho }}.$$

### 2.2. Asymmetric Structure

#### 2.2.1. Free Plate

**Figure 2:**
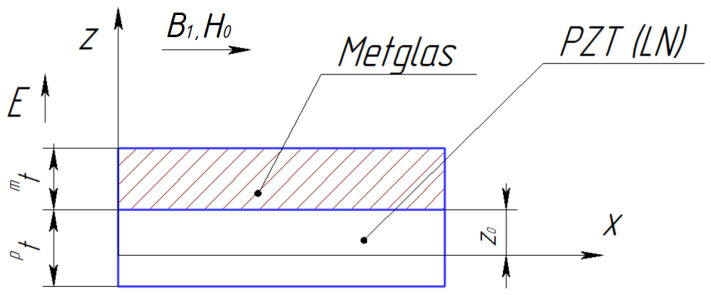
Asymmetric ME composite scheme.

Figure 2 shows the asymmetric ME composite’s structure for calculating the longitudinal and bending mode of the CME effect:

The CME coefficient in the resonant regime of the longitudinal mode can be found in Equation (21) for an asymmetric free ME composite, and only in Equations (1) and (2) is it necessary to remove the number 2 before tm. Also, it is necessary to remove the number 2 when integrating the thickness of the magnetostrictive phase in Equation (20).

#### 2.2.2. Rigid End of the Plate

**Figure 3:**
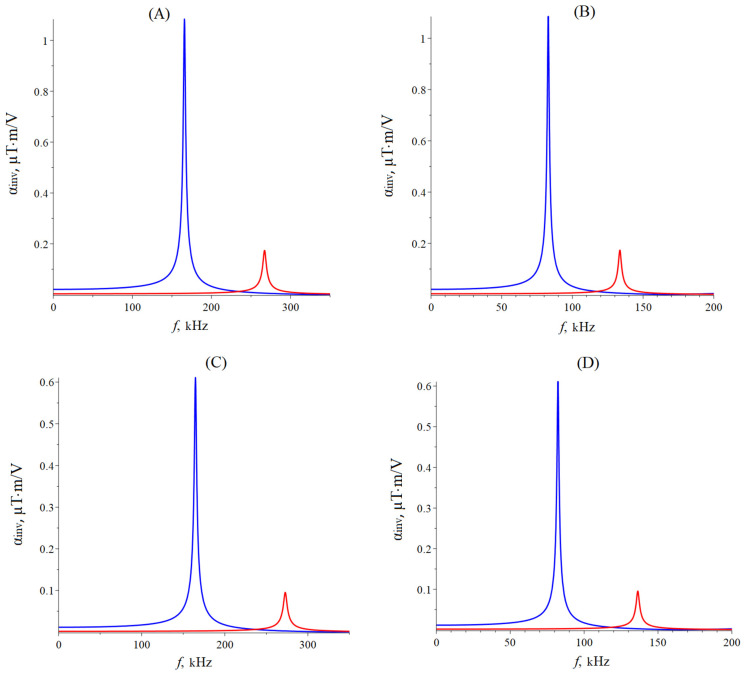
Dependence of the CME coefficient on the alternating electric field frequency applied to the ME composite’s piezoelectric phase for the longitudinal mode of the ME effect. (**A**) Free symmetric ME composite: the blue line is composite Metglas/PZT/Metglas, and the red line is composite Metglas/LN cut y + 128°/Metglas. (**B**) Symmetric ME composite with a rigidly fixed left end: the blue line is composite Metglas/PZT/Metglas, and the red line is composite Metglas/LN cut y + 128°/Metglas. (**C**) Free asymmetric ME composite: the blue line is composite Metglas/PZT, and the red line is composite Metglas/LN cut y + 128°. (**D**) Asymmetric ME composite with a rigidly fixed left end: the blue line is composite Metglas/PZT, and the red line is composite Metglas/LN cut y + 128°.

The CME coefficient in the resonant regime of the longitudinal mode can be found in Equation (28) for an asymmetric ME composite with a rigidly fixed left-end and the right one is free, and only in Equations (1) and (2) is it necessary to remove the number 2 before tm. Also, it is necessary to remove the number 2 when integrating the thickness of the magnetostrictive phase in Equation (27). Figure 3 below shows the dependence of the CME coefficient on the alternating electric field frequency applied to the ME composite’s piezoelectric phase for the longitudinal mode of the ME effect. The following parameters were used in the calculations: the magnetostrictive phase is Metglas with a thickness of tm = 29 µm; piezoelectric is PZT (LN cut y + 128°) with a thickness of tp = 0.5 mm; the resonance quality factor Q = 130; the length of the ME composite is 10 mm. Piezoelectric PZT is ceramic that is polarized along the thickness direction (the *Z*-axis is index 3). The Metglas width is equal to the piezoelectric width, and the ME composite width is much greater than its thickness; the ME composite’s width is less than its length. To take into account losses in the calculation, it is assumed $\omega =2\pi \left(1+\frac{1}{Q}i\right)f$. The resonant frequency calculated by Equation (22) for the longitudinal mode of the ME effect for the symmetric free ME composite Metglas/PZT/Metglas is 165.8 kHz; for the symmetric free ME composite, Metglas/LN cut y + 128°/Metglas is 267 kHz. The resonant frequency calculated by Equation (29) for the longitudinal mode of the ME effect for symmetric ME composite with rigidly fixed left-end Metglas/PZT/Metglas is 82.9 kHz, and for symmetric ME composite with rigidly fixed left-end Metglas/LN cut y + 128°/Metglas, it is 133.5 kHz. The resonant frequency, calculated by Equation (22) for the longitudinal mode of the ME effect for the asymmetric free ME composite Metglas/PZT is 164.6 kHz, and for the asymmetric free ME composite Metglas/LN cut y + 128°, it is 272.6 kHz. The resonant frequency calculated by Equation (29) for the longitudinal mode of the ME effect for asymmetric ME composite with rigidly fixed left-end Metglas/PZT is 82.3 kHz, and for asymmetric ME composite with rigidly fixed left-end Metglas/LN cut y + 128°, it is 136.3 kHz.

The CME coefficient value is two times less for an asymmetric ME composite for the longitudinal mode of the CME effect in comparison with a symmetric ME composite because an asymmetric ME composite has the volume of the magnetostrictive phase and is two times less than for a symmetric one. The resonant frequency of the CME effect for the longitudinal mode is greater for the composite with LN cut y + 128° as a piezoelectric than for a composite with PZT as a piezoelectric due to the lower effective density and greater effective stiffness of the composite with LN cut y + 128°. The CME coefficient value is greater for a composite with PZT as a piezoelectric than for a composite with LN cut y + 128°, which is due to a much larger piezoelectric coefficient PZT compared to LN cut y + 128° and a greater elastic compliance coefficient for the PZT than for LN cut y + 128°. The CME effect resonant frequency is two times less for the ME composite with a fixed left end than for the free ME composite, while the CME coefficient resonant value almost does not change.

## 3. EMR Bending Mode

The calculation is performed for an asymmetric ME composite (see Figure 2) for the bending mode in the EMR region.

### 3.1. Free Plate

The full thickness of the ME composite is

(30) t=tm+tp,

The longitudinal component of the strain tensor for magnetostrictive and piezoelectric phases is [47]

(31) Sp1=Sm1=S1=−z∂2w∂x2,

where w is the transverse displacement.

The longitudinal component of the magnetostrictive and piezoelectric phases stress tensor, electric field intensity’s third component applied to the piezoelectric phase of the ME composite, and the alternating magnetic induction in the magnetostrictive phase for $h_{1}=0$ are

(32) Tm1=YmS1Tp1=c11DS1−h31Dp3E3=−h31S1+β33SDp3Bm1=q11mT1,

where [47]

(33) c11D=sp11−d312ε33Tε0−1h31=c11Dd31ε33Tε0β33S=1+h31d31ε33Tε0,

$\varepsilon_{33}^{T}$ is the permittivity tensor’s component of the piezoelectric phase, and Dp3 is the piezoelectric phase’s electrical displacement.

The alternating magnetic induction in the magnetostrictive phase is determined by Equation (9).

The ME composite’s bending moment is

(34) M=∫z0−tpz0bzTp1dz+∫z0z0+tmbzTm1dz=−b∂2w∂x2D−btp2h31Dp3,

where b is the composite width,

(35) h31=1tp2∫z0−tpz0zh31dz=2z0−tp2tph31,

D=Dp+Dm is the full cylindrical stiffness of the composite [44],

(36) Dp=13c11Dtptp2−3tpz0+3z02Dm=13Ymtmtm2+3tmz0+3z02,

$z_{0}$ is the position of the boundary between the piezoelectric and magnetostrictive phases relative to the ME composite’s neutral line.

Let us find the electric voltage on the piezoelectric:

(37) U=∫z0−tpz0E3dz=tp2h31∂2w∂x2+tpβ33SDp3,

Find the electrical displacement in piezoelectric Dp3 from Equation (37) and substitute into Equation (34)

(38) M=−bt3c11∂2w∂x2−btph31β33SU,

where

(39) c11=1t3D−tp3h312β33S,

$z_{0}$ is determined from the minimum condition $\langle c_{11}\rangle$:

(40) z0=c11Dpt2−Ymmt2β33S−h312pt22Ymmt+c11Dptβ33S−h312pt,

The shear force is [47]:

(41) $$V=\frac{\partial M}{\partial x}=-bt^{3}\langle c_{11}\rangle \frac{\partial^{3}w}{\partial x^{3}},$$

The motion equation for bending vibrations of ME composite is [47]

(42) $$\rho bt\frac{\partial w^{2}}{\partial \tau^{2}}=\frac{\partial V}{\partial x},$$

Substitute Equation (41) for shear force into Equation (42):

(43) $$t^{2}\langle c_{11}\rangle \frac{\partial^{4}w}{\partial x^{4}}+\rho \frac{\partial w^{2}}{\partial \tau^{2}}=0,$$

The dependence of the displacement on time is harmonic $w∼e^{i\omega \tau }$, therefore:

(44) $$\begin{array}{l} \frac{\partial^{4}w}{\partial x^{4}}-k^{4}w=0\\ k={\left(\frac{\rho }{t^{2}\langle c_{11}\rangle }\omega^{2}\right)}^{\frac{1}{4}} \end{array},$$

where the composite effective density is determined by Equation (14).

The general solution to the motion equation is

(45) $$w=C_{1}\cosh\left(kx\right)+C_{2}\sinh\left(kx\right)+C_{3}\cos\left(kx\right)+C_{4}\sin\left(kx\right),$$

Then, we obtain

(46) $$S_{1}=-zk^{2}\left[C_{1}\cosh\left(kx\right)+C_{2}\sinh\left(kx\right)-C_{3}\cos\left(kx\right)-C_{4}\sin\left(kx\right)\right],$$

The equilibrium conditions for a free ME composite are

(47) $$\begin{array}{l} V\left(0\right)=0\\ M\left(0\right)=0\\ V\left(L\right)=0\\ M\left(L\right)=0 \end{array},$$

We obtain a linear inhomogeneous equation system for four unknowns $C_{1},\;C_{2},\;C_{3},\;C_{4}$ by substituting Equations (38) and (41) into Equation (47). We find four unknowns $C_{1},\;C_{2},\;C_{3},\;C_{4}$ from this equation system.

Then, substitute the obtained constants $C_{1},\;C_{2},\;C_{3},\;C_{4}$ into Equation (46) and find the alternating magnetic induction in the ME composite averaged over the magnetostrictive phase volume during the CME effect:

(48) B¯m1=1tL∫0L∫z0−tmz0Bm1dzdx=q11Ymh31Utptmtm−2z0r41−r1+r21−r34Lkt4c11β33Sr1r3−1,

where

(49) $$\begin{array}{l} r_{1}=\cosh\left(kL\right)\\ r_{2}=\sinh\left(kL\right)\\ r_{3}=\cos\left(kL\right)\\ r_{4}=\sin\left(kL\right) \end{array},$$

Then, the CME coefficient is calculated by the equation

(50) αinv=B¯m1tpU=q11Ymh31tp2tmtm−2z0r41−r1+r21−r34Lkt4c11β33Sr1r3−1.

The fundamental resonant frequency for this case is

(51) $$\begin{array}{l} f_{r}=\frac{\chi^{2}t}{2\pi L^{2}}\sqrt{\frac{\langle c_{11}\rangle }{\rho }}\\ \chi =4.73 \end{array}.$$

### 3.2. Rigid End of the Plate

The boundary conditions for the case when the left end of the ME composite is rigidly fixed and the right one is free:

(52) $$\begin{array}{l} w\left(0\right)=0\\ \frac{\partial w}{\partial x}\left(0\right)=0\\ V\left(L\right)=0\\ M\left(L\right)=0 \end{array},$$

The general solution to the motion equation corresponds to Equation (45).

After substituting Equations (45), (38) and (41) into Equation (52), we obtain a linear inhomogeneous system for determining unknown constants $C_{1},\;C_{2},\;C_{3},\;C_{4}$. Having found them from this system, we substitute them into Equation (46) and then find the alternating magnetic induction averaged over the volume of the ME composite during the CME effect:

(53) B¯m1=1tL∫0L∫z0−tmz0Bm1dzdx=q11Ymh31Utmtp2z0−tmr1r4+r2r32Lkt4c11β33Sr1r3+1.

Then, the CME coefficient is calculated by the equation

(54) αinv=B¯m1tpU=q11Ymh31tp2tm2z0−tmr1r4+r2r32Lkt4c11β33Sr1r3+1.

The fundamental resonant frequency for this case is

(55) $$\begin{array}{l} f_{r}=\frac{\chi^{2}t}{2\pi L^{2}}\sqrt{\frac{\langle c_{11}\rangle }{\rho }}\\ \chi =1.875 \end{array}.$$

**Figure 4:**
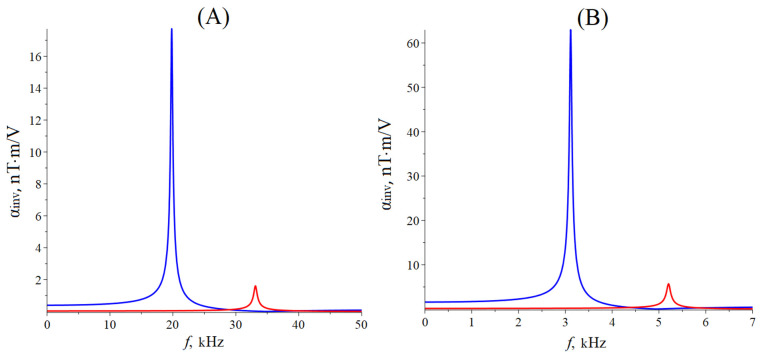
Dependence of the CME coefficient on the alternating electric field frequency applied to the ME composite’s piezoelectric phase for the bending mode of the ME effect. (**A**) Free asymmetric ME composite: the blue line is composite Metglas/PZT, and the red line is composite Metglas/LN cut y + 128°. (**B**) Asymmetric ME composite with a rigidly fixed left end: the blue line is composite Metglas/PZT, and the red line is composite Metglas/LN cut y + 128°.

Figure 4 below shows the dependence of the CME coefficient on the alternating electric field frequency applied to the ME composite’s piezoelectric phase for the bending mode of the ME effect. The ME composite’s material parameters are the same as for calculating the dependence of the CME coefficient on the alternating electric field frequency for the longitudinal mode.

The resonant frequency calculated by Equation (51) for the bending mode of the CME effect for the asymmetric free ME composite Metglas/PZT is 19.7 kHz, and for the asymmetric free ME composite Metglas/LN cut y + 128°, it is 33.1 kHz. The resonant frequency calculated by Equation (55) for the asymmetric ME composite with rigidly fixed left-end Metglas/PZT is 3.1 kHz, and for the asymmetric ME composite with rigidly fixed left-end Metglas/LN cut y + 128°, it is 5.2 kHz.

The CME effect resonant frequency for the bending mode is greater for a composite with the LN section y + 128° as a piezoelectric phase than for a composite with PZT due to the lower effective density and greater effective stiffness of the composite with LN cut y + 128°. The value of the CME coefficient is greater for a composite with PZT as a piezoelectric phase than for a composite with LN cut y + 128°, which is due to a much higher value of the piezoelectric coefficient PZT compared to LN cut y + 128°—a greater elastic compliance coefficient for the PZT than for LN cut y + 128°. The CME effect resonant frequency is three times less for the case when the composite’s left end is rigidly fixed than for the free ME composite, while the CME coefficient resonant value increases by more than three times for the bending mode of vibrations of the ME composite. The CME effect resonant frequency is much less for the bending mode than for the longitudinal mode for the free ME composite and the composite with the rigidly fixed left end, while the CME coefficient resonant values for the bending mode of vibrations are much less than for the longitudinal mode.

## 4. Quasi-Static Regime

When the bias field $H_{0}$ is oriented along the ME composite length, the CME coefficient in the quasi-static regime for a symmetric ME composite is determined by the contribution of only the longitudinal mode; for an asymmetric ME composite, the CME coefficient in the quasi-static regime is determined by the contribution of both the longitudinal and bending modes.

### 4.1. Symmetric Structure

Assuming in Equation (21) the frequency *f* is equal to zero, we obtain:

(56) αinv=2tmq11Ymνpd31tsp11c11,

### 4.2. Asymmetric Structure

Consider the motion equation for deformations:

(57) $$\rho \frac{\partial^{2}U_{x}}{\partial t^{2}}=\frac{\partial T_{1}}{\partial x},$$

Equation (57) has the form of the quasi-static regime:

(58) $$\frac{\partial T_{1}}{\partial x}=0,$$

This means that $T_{1}$ should not depend on *x*. This means that $S_{1}$ should also not depend on *x*. Since both the longitudinal and bending modes are excited in the asymmetric ME composite for the quasi-static regime, then

(59) $$S_{1}=A+zB.$$

The magnetostrictive phase’s material equations are

(60) Tm1=YmS1−q11h1,

(61) Bm1=μμ0h1+q11mT1,

Equations (60) and (61) have the form for $h_{1}=0$:

(62) Tm1=S1Ym,

(63) Bm1=q11mT1,

The longitudinal component of the piezoelectric phase stress tensor and electric field intensity third component, applied to the piezoelectric phase of the ME composite, are

(64) Tp1=c11DS1−h31D3,

(65) $$E_{3}=-h_{31}S_{1}+\beta_{33}^{S}D_{3},$$

The electrical voltage on the piezoelectric, taking into account that $D_{3}$ does not depend on z, is as follows:

(66) U=∫z0z0+tpE3dz=∫z0z0+tp−h31S1+β33SD3dz=∫z0z0+tp−h31A+zB+β33SD3dz=−h31tpA−12h31tp2z0+tpB+β33StpD3,

Let us express the electric displacement’s third component in the piezoelectric phase from Equation (66):

(67) D3=Utpβ33S+h31β33SA+122z0+tpB,

Substitute Equation (59) into Equation (62):

(68) Tm1=YmA+zB,

The first condition for the static equilibrium of an ME composite is that the total longitudinal force is equal to zero:

(69) F1=∫z0z0+tpTp1dz+∫z0−tmz0Tm1dz=β33SYmpt+c11Dmt−h312ptβ33SA+β33Sc11Dpttp+2z0+Ymtm2z0−tm−h312pt2z0+tp2β33SB−Uh31β33S=0,

The second condition for the static equilibrium of the ME composite is the zero total moment, which is given by:

(70) M1=∫z0z0+tpzTp1dz+∫z0−tmz0zTm1dz=β33Sc11Dpttp+2z0+Ymtm2z0−tm−h312pt2z0+tp2β33SA+4tpc11Dtp2+3z0tp+z0+4Ymtm3z02−3tmz0+tm2β33S−3h312pttp+2z02z0+tp12β33SB−tp+2z0Uh312β33S=0,

We use the well-known condition to determine *z*_0_, which is that the longitudinal force does not depend on the coefficient B associated with bending vibrations, and the bending moment does not depend on the coefficient A associated with longitudinal vibrations.

This condition is

(71) β33Sc11Dpttp+2z0+Ymtm2z0−tm−h312pt2z0+tp=0.

Then, we obtain

(72) z0=Ymmt2β33S−c11Dtp2β33S+h312tp22Ymmtβ33S+c11Dtpβ33S−h31tp.

**Figure 5:**
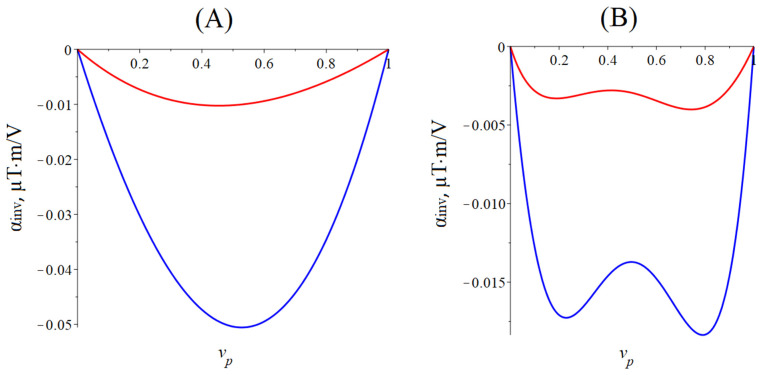
Dependence of the CME coefficient on the piezoelectric volume fraction in the quasi-static regime. (**A**) The blue line is the symmetric composite Metglas/PZT/Metglas, and the red line is the symmetric composite Metglas/LN cut y + 128°/Metglas. (**B**) The blue line is asymmetric composite Metglas/PZT, and the red line is asymmetric composite Metglas/LN cut y + 128°.

Let us determine coefficients A and B from Equations (69) and (70):

(73) A=−Uh31Ymtm3tm2z0−tp−4tm2+6z0tp−c11Dpt3Ym2tm4β33S−tmtpYmpt23h312−4c11Dβ33S+2h312−c11Dβ33S3tmtp+2tm2−c11Dpt4h312−c11Dβ33SB=6Uh31Ymtmtm+tpYm2tm4β33S−tmtpYmpt23h312−4c11Dβ33S+2h312−c11Dβ33S3tmtp+2tm2−c11Dpt4h312−c11Dβ33S,

Then, we find the alternating magnetic induction averaged over the volume of the ME composite B¯m1 during the CME effect, substituting in Equation (63) the obtained constants A and B; then, the CME coefficient is as follows:

(74) αinv=B¯m1tpU=tph31Ymq11tmYmtm3+c11Dpt3tYm2tm4β33S−tmtpYmpt23h312−4c11Dβ33S+2h312−c11Dβ33S3tmtp+2tm2−c11Dpt4h312−c11Dβ33S,

Figure 5 below shows the dependence of the CME coefficient on the piezoelectric volume fraction for the quasi-static regime of the CME effect. The material parameters of the ME composites are the same as for calculating the dependence of the CME coefficient on the alternating electric field frequency for the longitudinal mode of the CME effect.

The optimal piezoelectric volume fraction of a symmetric ME composite is 0.55, at which the CME coefficient is maximal for a composite with PZT and 0.45 for a composite with LN cut y + 128°. The optimal piezoelectric volume fraction is 0.8 for an asymmetric ME composite with PZT, and the optimal piezoelectric volume fraction is 0.75 for a composite with LN cut y + 128°. The CME coefficient value is greater for a composite with PZT as a piezoelectric phase than for a composite with LN cut y + 128°, which is due to a much larger piezoelectric coefficient PZT compared to LN cut y + 128°.

## 5. EMR Longitudinal Shear Mode

The equations for calculating the CME coefficient during the longitudinal shear mode are the same as for the longitudinal mode, but the longitudinal mechanical stress tensors are replaced by Tm1, Tp1 on the shear mechanical stress tensors Tm6,Tp6, the piezoelectric and piezomagnetic modulus $d_{31}$, $q_{11}$ are on $d_{36}$, $q_{16}$, the piezoelectric phase compliance coefficient sp11 is on sp66, the magnetostrictive phases Young’s modulus Ym is on shift modulus Gm, and the composite’s effective stiffness coefficient $c_{11}$ is on $c_{66}$.

**Figure 6:**
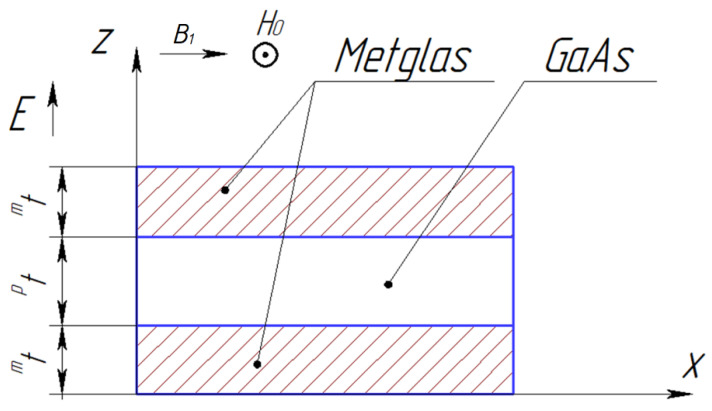
Symmetric ME composite scheme for the longitudinal shear mode.

Figure 6 shows the symmetric ME composite’s structure for calculating the longitudinal shear mode of the CME effect.

### 5.1. Symmetric Structure

The CME coefficient is calculated by the following equation:

(75) αinv=2tmq16Gmνpd36tgηsp66c66ηt,

where

(76) $$\eta =\frac{kL}{2},$$

(77) $$k=\sqrt{\frac{\rho }{c_{66}}}\omega ,$$

(78) ρ=νmρm+νpρp,

(79) c66=νpsp66+νmGm,

The fundamental resonant frequency for this case is [47]

(80) $$f_{r}=\frac{1}{2L}\sqrt{\frac{c_{66}}{\rho }}.$$

### 5.2. Asymmetric Structure

The CME coefficient can be found in Equation (75) for an asymmetric ME composite in the resonant mode of the longitudinal-shear ME mode for a free composite, and it is necessary to remove the number 2 before tm in Equations (1), (2) and (75).

## 6. EMR Torsional Mode for ME Composite of Metglas/GaAs

**Figure 7:**
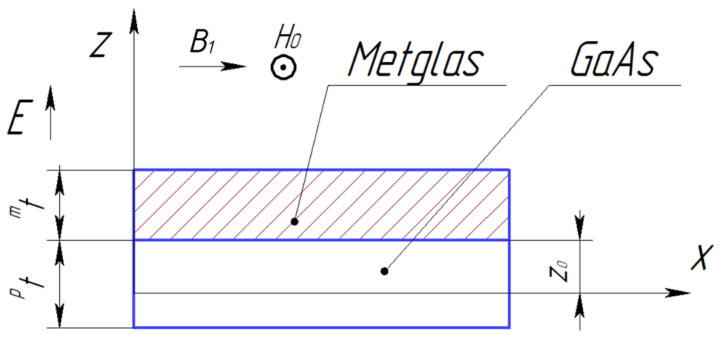
Asymmetric ME composite scheme for the torsional mode.

The calculation is performed for an asymmetric ME composite (see Figure 7) for the torsional mode in the EMR region.

The orientations of the crystallographic axes are [100], [010], and [001], where [100] is the length (*X*-axis is index 1), [010] is the width (*Y*-axis is index 2), and [001] is a thickness (*Z*-axis is index 3), respectively, for GaAs as a ME composite’s piezoelectric.

The *X*-axis is drawn along the composite length in the corresponding symmetry plane of the composite, along the composite beam rotation axis during torsional vibrations in the direction of the composite length. The *Y*-axis will be directed along the composite width. The bias field $H_{0}$ is directed along the *Y*-axis, and the alternating electric field Ep3 is directed along the *Z*-axis.

The full thickness of the ME composite is determined by Equation (30).

The ME composite’s strain tensor shear components are as follows:

(81) $$\begin{array}{l} S_{5}=y\frac{\partial \theta }{\partial x}\\ S_{6}=-z\frac{\partial \theta }{\partial x} \end{array},$$

where θ is the twist angle.

The material equation for the piezoelectric phase is

(82) S5=1GpTp5S6=1GpTp6+d36Ep3,

where Gp is the piezoelectric phase’s shift modules.

We find the piezoelectric phase’s stress tensor tangent components from Equation (82):

(83) pT5=GpS5=Gpy∂θ∂x,

(84) pT6=GpS6−d36Ep3=−Gpz∂θ∂x−d36GpEp3,

The material equations of the magnetostrictive phase for $h_{1}=0$ as follows:

(85) S5=1GmTm5S6=1GmTm6,

We find the magnetostrictive phase’s stress tensor tangent components from Equation (85):

(86) mT5=GmS5=Gmy∂θ∂xmT6=GmS6=−Gmz∂θ∂x,

The ME composite’s magnetic induction for $h_{1}=0$ is:

(87) Bm1=q16mT6,

The electric displacement in piezoelectric is:

(88) D3=d36Tp6+εε0Ep3=−d36pGz∂θ∂x+εε0−Gpd362Ep3,

where ε is the piezoelectric phase’s permittivity.

We express Ep3 from Equation (88):

(89) Ep3=h36z∂θ∂x+β33SD3,

where

(90) h36=d36Gpεε0−Gpd362β33S=1εε0−Gpd362,

and substitute it into Equation (84). As a result, we obtain

(91) pT6=−GpDz∂θ∂x−h36D3,

where

(92) GpD=εε0Gpεε0−Gpd362.

The torque is as follows:

(93) M=∫−b2b2dy∫z0−tpz0yTp5−zTp6dz+∫−b2b2dy∫z0z0+tmyTm5−zTm6dz=∫−b2b2dy∫z0−tpz0yGpy∂θ∂x−z−zGpD∂θ∂x−h36D3dz+∫−b2b2dy∫z0z0+tmyGmy∂θ∂y+zGmz∂θ∂ydz=K0∂θ∂y+bh36tp2D3,

where

(94) K0=Kp+KmKp=13GpDz03−z0−tp3b+112Gptpb3Km=13Gmz0+tm3−z03b+112Gmtmb3h36=1tp2∫z0−tpz0zh36dz=h362z0−tp2tp,

where $z_{0}$ is the position of the boundary between the piezoelectric and magnetostrictive phases relative to the ME composite’s rotation axis.

Let us find the electric voltage on the piezoelectric:

(95) U=∫z0−tpz0Ep3dz=∫z0−tpz0h36z∂θ∂x+β33SD3dz=tp2h36∂θ∂x+tpβ33SD3,

We express the electric displacement in a piezoelectric from Equation (95)

(96) D3=Utpβ33S−tph36β33S∂θ∂x

and substitute it into Equation (93):

(97) M=−bt3G∂θ∂x−btph36β33SU,

where [47]

(98) G=1bt3K0−btp3h362β33S.

$z_{0}$ is determined from the condition of the minimum effective shear modulus of the ME composite $\langle G\rangle$:

(99) z0=GpDpt2β33S−Gmmt2β33S−h362pt22Gmmtβ33S+GpDptβ33S−h362pt,

The torsional vibrations are as follows:

(100) $$J\frac{\partial \theta^{2}}{\partial \tau^{2}}=\frac{\partial M}{\partial x},$$

where the ME composite’s moment of inertia per unit width is

(101) J=ρpIp+ρmIm,

where the piezoelectric and magnetostrictive phases polar moments are

(102) Ip=tpb13tp2−tpz0+z02+112b2Im=tmb13tm2+tmz0+z02+112b2,

Substitute Equation (97) into Equation (100):

(103) $$J\frac{\partial^{2}\theta }{\partial \tau^{2}}=-bt^{3}\langle G\rangle \frac{\partial^{2}\theta }{\partial x^{2}},$$

The dependence of the twist angle on time is harmonic $\theta ∼e^{i\omega \tau }$, therefore:

(104) $$\frac{\partial^{2}\theta }{\partial x^{2}}+k^{2}\theta =0,$$

where the wave number is

(105) $$k=\omega \sqrt{\frac{J}{bt^{3}\langle G\rangle }}.$$

The general solution to Equation (104) is as follows:

(106) $$\theta =A\cos\left(kx\right)+B\sin\left(kx\right).$$

The equilibrium conditions for a free ME composite are

(107) $$\begin{array}{l} M\left(\frac{L}{2}\right)=0\\ M\left(-\frac{L}{2}\right)=0 \end{array},$$

We obtain a linear inhomogeneous equation system for two unknowns A and B by substituting Equation (97) into Equation (107). Then, we find two unknowns A and B, from this equation system and substitute them into Equation (87). After that, we find the alternating magnetic induction occurring in the magnetostrictive phase B¯m1 during the CME effect. And we will find the CME coefficient:

(108) αinv=B¯m1tpU=q16Gmh36tp2tm2z0+tm2ηt4Gβ33Stgη,

where

(109) $$\eta =\frac{kL}{2}.$$

The fundamental resonant frequency for this case is

(110) $$f_{r}=\frac{1}{2L}\sqrt{\frac{bt^{3}\langle G\rangle }{J}}.$$

**Figure 8:**
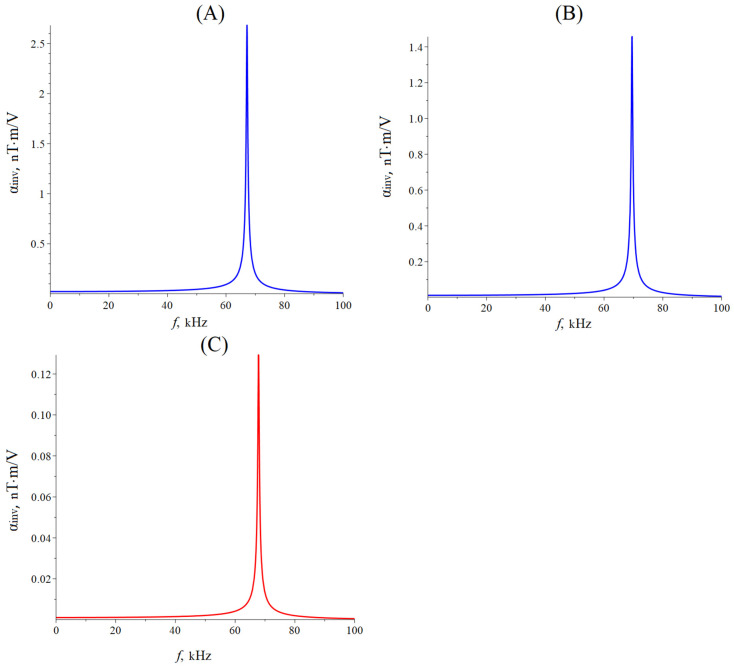
Dependence of the CME coefficient on the alternating electric field frequency applied to the free ME composite’s piezoelectric phase for the longitudinal-shear and torsional modes. (**A**) Longitudinal shear mode, a symmetric free ME composite Metglas/GaAs/Metglas; (**B**) longitudinal-shear mode, an asymmetric free ME composite Metglas/GaAs; (**C**) torsional mode, a symmetric free ME composite Metglas/GaAs.

Figure 8 below shows the dependence of the CME coefficient on the alternating electric field frequency applied to the ME composite’s piezoelectric phase for the longitudinal-shear and torsional modes.

The magnetostrictive phase is Metglas, with a thickness of ${}^{m}t$= 29 µm. The piezoelectric is GaAs with a thickness of ${}^{p}t$= 0.2 mm. The value of the quality factor Q= 300. The ME composite length was 23 mm, and the width was 0.3 mm.

The resonant frequency calculated by Equation (80) for the longitudinal shear mode of the CME effect for the symmetric free ME composite Metglas/GaAs/Metglas is 67.1 kHz, and for the asymmetric free ME composite Metglas/GaAs, it is 69.5 kHz. The resonant frequency, calculated by Equation (110) for the torsional mode of the CME effect for the asymmetric free ME composite Metglas/GaAs, is 67.8 kHz.

The resonant frequency of the CME effect is slightly less for the longitudinal shear mode for an asymmetric ME composite with GaAs as a piezoelectric phase than for the torsional mode, while the resonant value of the CME coefficient is more than ten times greater for the longitudinal shear mode than for the torsional mode. The value of the CME coefficient is almost two times less for an asymmetric ME composite for the longitudinal shear mode in comparison with a symmetric ME composite because the volume of the magnetostrictive phase is two times less for an asymmetric ME composite than for a symmetric one.

It was determined in previous studies that in an asymmetric ME composite, with the orientation of the bias field shown in Figure 6, both longitudinal shear and torsional modes exist simultaneously during the CME effect. Moreover, the resonant frequency of the longitudinal shear mode and the torsional mode differ slightly, and the CME coefficient’s value for the longitudinal shear mode is an order of magnitude greater than for the torsional mode. Because of this, the contribution of the torsional mode to the full CME coefficient is insignificant against the background of the longitudinal shear mode, and it is difficult to determine experimentally. One of the possible ways out of this situation may be the use of a bimorph LN Zy + 45° as a piezoelectric phase in the ME composite.

## 7. EMR Torsional Mode in a ME Composite Based on Bimorph LN

The *X*-axis is drawn along the composite length in the corresponding symmetry plane of the composite, along the composite beam rotation axis during torsional vibrations in the direction of the composite length. The *Y*-axis will be directed along the composite width. The bias field $H_{0}$ is directed along the *Y*-axis, and the alternating electric field Ep3 is directed along the *Z*-axis.

**Figure 9:**
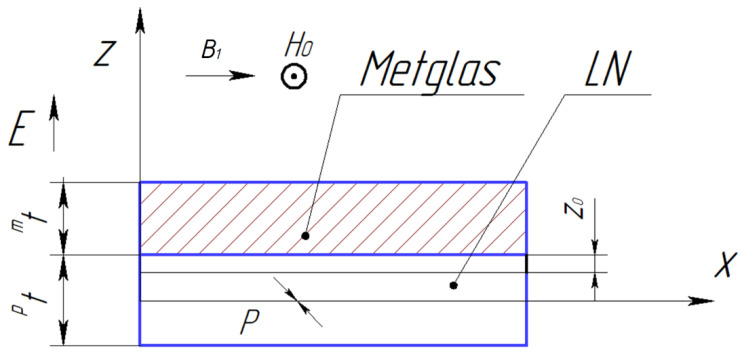
Asymmetric ME composite scheme with bimorph LN Zy + 45° phase.

Figure 9 shows the asymmetric ME composite’s structure with bimorph LN Zy + 45° as a piezoelectric phase for calculating the torsional mode of the CME effect:

A bimorph LN Zy + 45° consists of two LN Zy + 45° plates having the opposite polarization direction along the thickness. The crystallographic axis, corresponding to the thickness of the bimorph layer for one plate, is [101] (the *Z*-axis is index 3), and for the other plate, it is [−10−1]. Crystallographic axes, directed along the length and width, are [10−1] (the *X*-axis is index 1) and [010] (the *Y*-axis is index 2), respectively.

The ME composite’s strain tensor shear components are determined by Equation (81).

The material equations of the magnetostrictive phase for $h_{1}=0$ correspond to Equations (86) and (87).

(111) Tp5=cp55ES5+cp56ES6−e35Ep3Tp6=cp56ES5+cp66ES6−e36Ep3D3=e35S5+e36S6+ε33ε0Ep3,

where cp55E, cp56E, cp66E are the shear components at a constant electric field intensity of the piezoelectric phase’s stiffness tensor, and $e_{35},\;\;e_{36}$ are the piezoelectric coefficients at a constant electric field intensity.

Let us express Tp5,Tp6,Ep3:

(112) Tp5=cp55DS5+cp56DS6−h35D3Tp6=cp56DS5+cp66DS6−h36D3Ep3=−h35S5−h36S6+β33SD3=−h35y∂θ∂x+h36z∂θ∂x+β33SD3,

where cp55D, cp56D, cp66D are the shear components at a constant electrical displacement of the piezoelectric phase’s stiffness tensor, and $h_{35},\;\;h_{36}$ are the piezoelectric coefficients for the constant strain tensor shear components.

(113) cp55D=cp55E+e352ε33ε0cp66D=cp66E+e362ε33ε0cp56D=cp56E+e35e36ε33ε0h35=e35ε33ε0h36=e36ε33ε0β33S=1ε33ε0,

The torque is as follows:

(114) M=Mp+Mm=∫−b2b2dy∫z0−tpz0yTp5−zTp6dz+∫−b2b2dy∫z0z0+tmyTm5−zTm6dz=∫−b2b2dy∫z0−tpz0yc55DS5+cp56DS6−h35D3−zcp56Dy∂θ∂x−cp66Dz∂θ∂x−h36D3dz+∫−b2b2dx∫z0z0+tmyGmy∂θ∂x+zGmz∂θ∂xdz=Q˜∂θ∂x+btp2h36D3,

where

(115) Q˜=Qp+Qm,

where $z_{0}$ is the position of the interface between the piezoelectric and magnetostrictive phases relative to the composite beam rotation axis.

We have tp1=tp2=tp/2, hp236=−hp136=h36 for bimorph LN Zy + 45°, where

(116) h36=1tp2∫z0−tp2−tp1z0zh36dz=∫z0−tp2−tp1z0−tp1zhp236dz+∫z0−tp1z0zhp136dz=12tp2hp236tp22z0−2tp1−tp2+hp136tp12z0−tp1=14hp136,

The piezoelectric and ferromagnet phases’ polar moments of the shear stiffness coefficient are given by:

(117) Qp=tpbcp66D13tp2−tpz0+z02b+112cp55Db2Qm=Gmtmb112b2+13tm2+tmz0+z02,

Let us find the electric voltage on the piezoelectric:

(118) U=∫z0−tpz0Ep3dz=∫z0−tpz0−h35y∂θ∂x+h36z∂θ∂x+β33SD3dz=tp2h36∂θ∂x+tpβ33SD3,

We express the electric displacement in a piezoelectric as

(119) D3=Utpβ33S−tph36β33S∂θ∂x,

Substitute Equation (119) into Equation (114):

(120) M=Q∂θ∂x+btph36β33SU,

where

(121) Q=Q˜−btp3h362β33S,

The position of the interface between the piezoelectric and magnetostrictive phases relative to the composite beam rotation axis $z_{0}$ is determined from the condition of the minimum polar moments of the shear stiffness coefficient Q:

(122) ∂Q∂z0=0z0=cp66Dpt2−cm44mt22cm44mt+cp66Dpt,

The torsional vibrations are determined by Equation (100), the ME composite’s moment of inertia per unit width is determined by Equation (101), where the piezoelectric and magnetostrictive phases polar moments are determined by Equation (102).

Substitute Equation (120) into Equation (100):

(123) $$J\frac{\partial^{2}\theta }{\partial \tau^{2}}=Q\frac{\partial^{2}\theta }{\partial x^{2}},$$

The dependence of the twist angle on time is harmonic $\theta ∼e^{i\omega \tau }$, therefore:

(124) $$Q\frac{\partial^{2}\theta }{\partial x^{2}}+J\omega^{2}\theta =0,$$

(125) $$\frac{\partial^{2}\theta }{\partial x^{2}}+k^{2}\theta =0,$$

where the wave number is

(126) $$k=\omega \sqrt{\frac{J}{Q}},$$

The general solution of Equation (125) is determined by Equation (106). The equilibrium conditions for a free ME composite correspond to Equation (107).

We obtain a linear inhomogeneous equation system for two unknowns A and B by substituting Equation (120) into equilibrium conditions for a free ME composite (107). Solving this system, we find A and B. Then, we substitute the found constants into Equation (87) and find the alternating magnetic induction occurring in the magnetostrictive phase B¯m1 during the CME effect. After that, we will find the CME coefficient:

(127) αinv=B¯m1tpU=q16Gmh36btp2tm2z0+tm2ηQβ33Sttgη,

where

(128) $$\eta =\frac{kL}{2},$$

The fundamental resonant frequency for this case is

(129) $$f_{r}=\frac{1}{2L}\sqrt{\frac{Q}{J}}.$$

**Figure 10:**
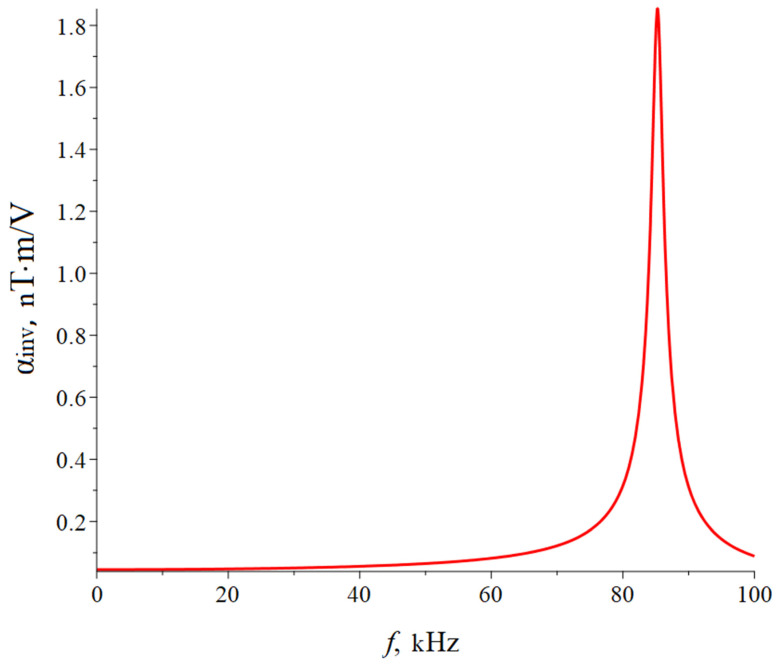
Dependence of the CME coefficient on the alternating electric field frequency applied to the free ME composite’s piezoelectric phase for the torsional mode. Asymmetric free ME composite bimorph LN Zy + 45°/Metglas.

Figure 10 below shows the dependence of the CME coefficient on the alternating electric field frequency applied to the ME composite’s piezoelectric phase for the longitudinal-shear and torsional modes for a composite with a bimorph LN Zy + 45°.

The following material parameters were used in the calculation: The length of the ME composite was L = 23 mm, and the width was b = 0.5 mm. In the calculation, the following material parameters of the initial components were used: For Metglas: ρm = 7180 kg/m^3^, Gm = 3.85 × 10^10^ Pa, $q_{16}$ = 1.0 × 10^−9^ m/A, and ${}^{m}t$ = 29 µm. For LN Zy + 45°: ρp = 4647 kg/m^3^, cp55E = 6.75 × 10^10^ Pa, cp56E = 7.5 × 10^9^ Pa, cp66E = 6.75 × 10^10^ Pa, $\varepsilon_{33}$ = 36.5, ep135 = −ep235 = 2.5 C/m^2^, ep136 = −ep236 = 2.5 C/m^2^, and ${}^{p}t$ = 0.4 mm. The value of the quality factor for this ME composite Q = 100.

The resonant frequency calculated by Equation(129) for the torsional mode of the CME effect for the asymmetric free ME composite bimorph LN Zy + 45°/Metglas is 85.2 kHz.

The value of the CME coefficient is much greater for the torsional mode for an asymmetric composite with bimorph LN Zy + 45° as a piezoelectric phase than for a composite with GaAs.

The longitudinal shear mode of the CME effect in an asymmetric composite with a bimorph LN will not be excited. If some type of CME effect is observed in the experiment, then it can only occur from the torsional mode. Thus, the longitudinal shear mode interfering with the observation of the torsional mode will be eliminated.

## 8. Quasi-Static Regime

When the bias field $H_{0}$ is oriented along the ME composite width, the CME coefficient in the quasi-static regime for a symmetric ME composite is determined by the contribution of only the longitudinal shear mode; for an asymmetric ME composite, the CME coefficient in the quasi-static regime is determined by the contribution of both the longitudinal shear and torsional modes.

### 8.1. ME Composite of Metglas/GaAs

#### 8.1.1. Symmetric Structure

Assuming in Equation (75) the frequency f is equal to zero, we obtain:

(130) αinv=2tmq16Gmνpd36sp66c66t,

#### 8.1.2. Asymmetric Structure

There are no vibrations in the direction of the length of the composite in the quasi-static regime. This means that $S_{5}$ and $S_{6}$ should not depend on *x*. Since both the longitudinal shear and torsional modes are excited in the asymmetric ME composite for the quasi-static regime as follows:

(131) $$\begin{array}{l} S_{5}=yB\\ S_{6}=A-zB \end{array},$$

Substitute Equation (131) into Equations (83), (84), and (86):

(132) mT5=GmyBmT6=GmA−GmzB,

(133) pT5=GpyBpT6=GpA−zB−d36Ep3,

The electric displacement in a piezoelectric is

(134) D3=d36Tp6+εε0Ep3=d36pGA−zB+εε0−Gpd362Ep3,

Let us express electric field intensity in the piezoelectric from Equation (134)

(135) Ep3=β33SD3−h36A−zB

and substitute it into Equation (133). As a result, we obtain

(136) pT6=GpDA−zB−h36D3.

The electric voltage on the piezoelectric is

(137) U=∫z0−tpz0Ep3dz=tpβ33SD3−h36tpA−B2z0−tp2.

Then, the electric displacement in the piezoelectric, expressed in terms of U is

(138) D3=Utpβ33S+h36β33SA−B2z0−tp2.

The first condition for the static equilibrium of the ME composite is the total tangential force on the site perpendicular to the *X*-axis along the *Y*-axis, and is equal to zero and is given by the following equation:

(139) ∫z0−tpz0Tp6dz+∫z0z0+tmTm6dz=0,

The second condition for the static equilibrium of the ME composite is the zero torque and is given by:

(140) ∫−b2b2dy∫z0−tpz0yTp5−zTp6dz+∫−b2b2dy∫z0z0+tmxTm5−zTm6dz=0,

We use the well-known condition to determine $z_{0}$, which is that the longitudinal force does not depend on the coefficient B associated with the torsional vibrations, and the bending moment does not depend on the coefficient A associated with longitudinal-shear vibrations.

This condition is as follows:

(141) GpDtp2z0−tp+Gmtm2z0+tm=0,

Then, we obtain

(142) z0=pt2GpD−mt2Gm2mtGm+tpGpD.

Equations (139) and (140) form a linear inhomogeneous system of two equations with two unknowns A and B. Solving it, we find the unknowns A and B.

Magnetic induction of the ME composite’s magnetostrictive phase for $h_{1}=0$ is as follows:

(143) Bm1=q16GmA−zB.

Then, we find the alternating magnetic induction averaged over the ME composite volume in the magnetostrictive phase during the CME effect, substituting into Equation (143) the obtained constants A and B. Then, we will determine the CME coefficient:

(144) αinv=B¯m1tpU=tptmGmq16h36tGpDβ33S−h362tp4GpD+tp2b2Gp+6tm2Gm+Gmtp3tm4GpDβ33S−3h362⋅tp3GpD+b2tpGp+tmGmb2+tm2+Gmtptm4tm2GpDβ33S−h362+b2Gp+GpDβ33S−h362+β33Stm2Gm2b2+tm2,

### 8.2. ME Composite Based on Bimorph LN

Assuming in Equation (127) the frequency *f* is equal to zero, we obtain:

(145) αinv=q16Gmh36btp2tm2z0+tm2Qβ33St.

**Figure 11:**
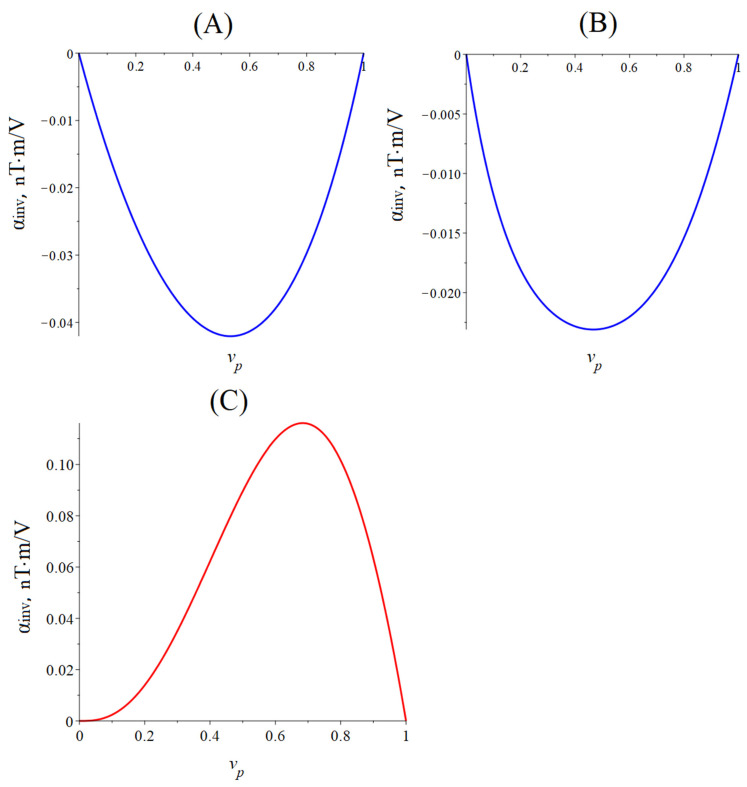
Dependence of the CME coefficient on the piezoelectric volume fraction in the quasi-static regime. (**A**) Symmetric ME composite Metglas/GaAs/Metglas; (**B**) asymmetric ME composite Metglas/GaAs; (**C**) asymmetric ME composite bimorph LN Zy + 45°/Metglas.

Figure 11 below shows the dependence of the CME coefficient on the piezoelectric volume fraction for the quasi-static regime of the CME effect for longitudinal-shear and torsional modes. The material parameters of the ME composites are the same as for calculating the dependence of the CME coefficient on the alternating electric field frequency in the EMR region.

The optimal piezoelectric volume fraction of a symmetric ME composite is 0.54, at which the CME coefficient is maximal for a composite with GaAs as piezoelectric and 0.45 for an asymmetric ME composite with GaAs. The optimal piezoelectric volume fraction is 0.68 for an asymmetric ME composite with bimorph LN Zy + 45°. The maximum value of the CME coefficient is approximately five times greater for an asymmetric ME composite with a bimorph LN Zy + 45° than for an asymmetric ME composite with GaAs as a piezoelectric in the quasi-static regime.

## 9. Discussion

We would first like to discuss the applicability limits of our theoretical calculation of the CME coefficient in this section. One of our main assumptions is that we consider the mechanical coupling between the magnetostrictive and piezoelectric phases to be ideal. Therefore, we consider the corresponding strain tensor components of the magnetostrictive and piezoelectric phases to be equal. In practice, the mechanical coupling between the magnetostrictive and piezoelectric phases may be quite far from ideal. This is due to the methods of connecting the phases: gluing or some other methods. At the same time, the mechanical deformation effect is transferred from the piezoelectric phase to the magnetostrictive one and is not transferred as well as it is described by theory during the CME effect in the experiment. Therefore, the experimental CME coefficient is always less than the theoretical one. Another assumption we use in modeling is that we consider the alternating magnetic field inside the magnetostrictive phase to be zero. This assumption justifies itself well when the ME composite length is greater than its width and the width is significantly greater than its thickness. In fact, this is not exactly true. A more accurate picture could be given by finite element modeling in such a software package as Comsol Multiphysics 6.2 for the alternating magnetic field distribution inside and around the magnetostrictive phase. Another assumption close in meaning is that we consider the electric induction vector’s component directed along the ME composite’s thickness independent of the coordinate along the thickness when considering bending and torsional modes. Again, this assumption works well with a long, narrow, very thin ME composite. However, a more accurate calculation could be made by the method of finite element modeling in Comsol Multiphysics. It is also necessary to discuss the complexity of observing the CME effect’s torsional mode against the background of the CME effect’s longitudinal shear mode. These modes exist simultaneously in an asymmetric ME composite when the CME effect is excited by applying an alternating electric voltage to the piezoelectric phase. Calculations show that the CME effect’s main resonant frequency for the longitudinal shear mode for an asymmetric ME composite with GaAs as a piezoelectric phase is slightly less than for the torsional mode, while the resonant value of the CME coefficient is more than ten times greater for the longitudinal shear mode than for the torsional mode. Therefore, it is difficult to observe such a small CME effect from the torsional mode against the background of a relatively large CME effect from the longitudinal shear mode in a small general frequency range. One of the possible ways to solve this problem is to use bimorphic LN Zy + 45° with the same LN layer thickness with oppositely directed polarization along the ME composite’s thickness instead of GaAs. The theory shows that in the ME composite based on the bimorphic LN Zy + 45°, the longitudinal shear mode is not excited. Therefore, the observation of the CME effect’s torsional mode will be greatly simplified. Also, we would like to explain why the article does not compare our theoretical calculations with the experimental data of other authors. This is because we consider relatively simple ME structures with the same length and width as the magnetostrictive and piezoelectric phases in modeling. Other authors use more complex ME structures in their experiments, in which the lengths or widths of the magnetostrictive and piezoelectric phases do not coincide [34,35,40,48,49,50], and other additional layers are present in the design; for example, a permanent magnet [51]. Such complex constructions of ME composites require separate, rather laborious calculations for comparison with our theory when observing the CME effect.

## 10. Conclusions

The general theory of the CME effect in composites in the low-frequency range, including the EMR region, is presented. The main EMR modes are considered in more detail, such as longitudinal, bending, longitudinal-shear, and torsional. To demonstrate the theory, expressions for the CME coefficients were obtained for symmetric and asymmetric GaAs/Metglas and LN/Metglas layered structures. To complete the analysis, a brief overview of the main works on the CME effect in the low-frequency range, including in the field of EMR, is given.

This article notes a number of limitations of the presented theory of the CME effect that must be taken into account. This is, first of all, the imperfection of the mechanical connection between the piezoelectric and magnetostrictive phases associated with the commonly used adhesive technology. The following restrictions are due to the presence of an alternating magnetic field inside the magnetostrictive phase and the constant direction of the electrical induction vector along the thickness of the composite in the case of bending and torsional modes.

Recommendations are given for the experimental study of the CME effect in the torsional mode in a bimorph structure based on Metglas/LN, which will eliminate the longitudinal shear mode and study only the spectrum of the torsional mode. To reliably calculate the CME effect, taking into account the complexity of the problem, it is proposed to use our analytical estimates presented in this article and compare them with calculations based on the Comsol Multiphysics package and the results of the experiments.

## Data Availability

Data are contained within the article.
